# Zeroing In on a Risk Factor? PBDE Exposure and Acute Lymphoblastic Leukemia

**DOI:** 10.1289/ehp.122-A282

**Published:** 2014-10-01

**Authors:** Tanya Tillett

**Affiliations:** Tanya Tillett, MA, of Durham, NC, is a staff writer/editor for *EHP*. She has been on the *EHP* staff since 2000 and has represented the journal at national and international conferences.

Although no longer manufactured in the United States, polybrominated diphenyl ether (PBDE) flame retardants are still found in imported products as well as in older U.S.-made products; they also persist in the environment.^1^ A new study reported this month in *EHP* suggests that exposure to specific PBDE congeners may be a risk factor for acute lymphoblastic leukemia (ALL).^2^

ALL is the most common childhood leukemia in most Western countries, and it typically is diagnosed in children between 2 and 5 years of age.^3^ “We were interested in evaluating whether chemicals found in and around the home might be risk factors for childhood leukemia,” says study coauthor Mary Ward, an epidemiologist at the National Cancer Institute. Ward and colleagues are assessing such risks through the Northern California Childhood Leukemia Study, a case–control study conducted between 1995 and 2008. In another finding from the same study, higher levels of polychlorinated biphenyls (PCBs) in house dust were associated with an increased risk of ALL in resident children.^4^

**Figure:**
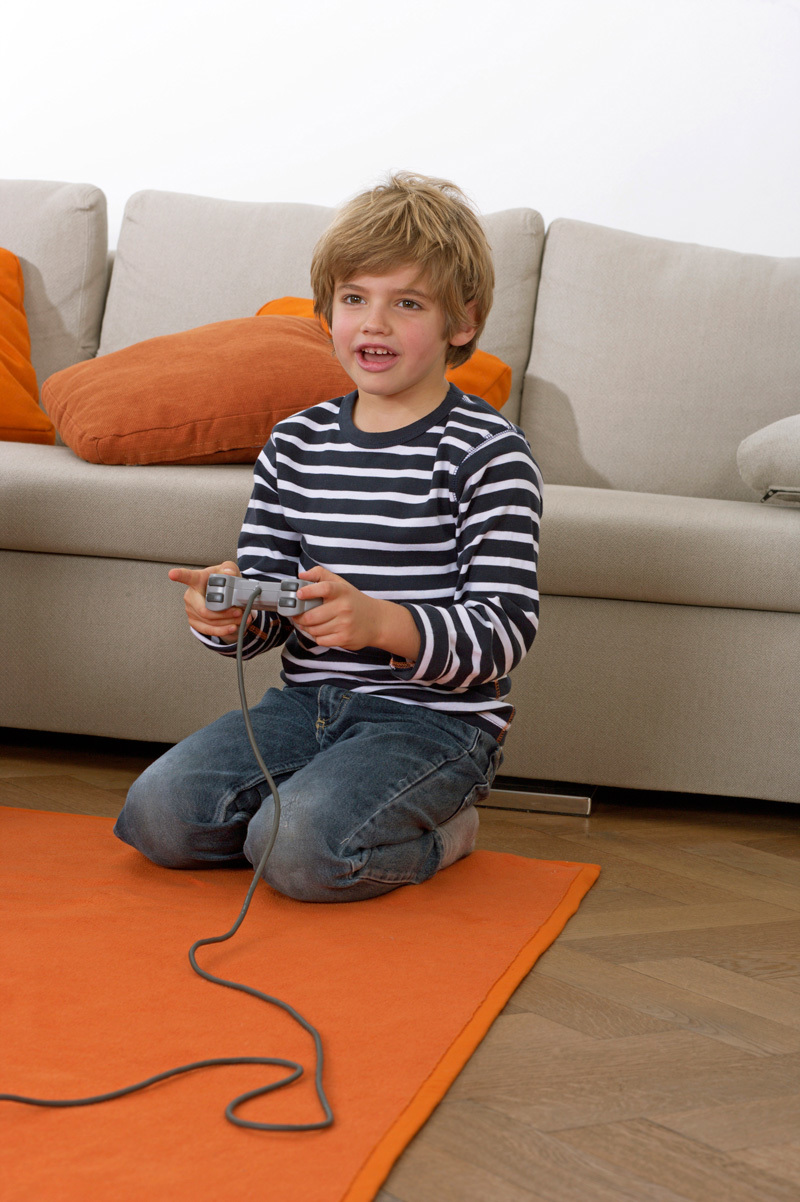
Carpets and rugs can become repositories for chemicals shed by upholstered furniture, electronics, and other consumer goods. © Kay Blaschke/Getty

Commercially available PBDE formulations included penta-, octa-, and decaBDE, each comprising several different constituent congeners. PBDEs are structurally similar to PCBs and, like PCBs, accumulate in dust in the indoor environment.^2^ House dust is believed to be an important source of exposure to PBDEs, and exposures may be particularly high for toddlers and children, who ingest more dust due to frequent hand-to-mouth activity.^5^

The current study included 167 children with ALL, ranging in age from 0 to 7 years, and 214 matched controls. For each child, the investigators collected a sample of dust from the room where the child spent the most time while awake. These samples were analyzed for PBDE content.

The researchers found no association between total PBDE exposure and risk of ALL. But children living in homes with the highest concentrations of specific congeners—less common octa- and nonaBDEs with 8 and 9 bromine atoms—were more likely to be ALL cases than children living in homes with the lowest or undetectable levels of the same congeners.^2^

These less common congeners have thus far escaped scrutiny. “Up until a few years ago, nobody was even measuring them,” says Linda Birnbaum, director of the National Institute of Environmental Health Sciences. “The fact that exposure to these higher-brominated PBDEs is associated with higher cancer risk should raise a concern.”

To date, there is limited information on the toxicities of octa- and nonaBDEs. BDE-209, the major constituent of decaBDE, is the only PBDE to have been studied for carcinogenicity in animals.^6^ The U.S. Environmental Protection Agency deemed the results suggestive of carcinogenic potential and worthy of further exploration.^1^ The National Toxicology Program is currently conducting long-term carcinogenicity studies of pentaBDE and its major constituents.^7^

Given that childhood leukemia is relatively rare, the new study was limited by its sample size. It also was limited by measuring PBDEs only in dust samples and using a single dust sample as a basis for characterizing exposure. “It would be helpful to also analyze the PBDE levels in the blood of mothers and their children,” Birnbaum comments.

These preliminary results must be replicated in other populations before any conclusions can be drawn about the relationship between PBDEs and ALL. In the meantime, Birnbaum has good news. “Since the ban on PBDEs in the U.S., we are seeing their levels go down in the population,” she says.^8^ She predicts PBDE levels will continue to decline as older products containing them are phased out of use.
